# The Effectiveness of Zinc Supplementation in Taste Disorder Treatment: A Systematic Review and Meta-Analysis of Randomized Controlled Trials

**DOI:** 10.1155/2023/6711071

**Published:** 2023-03-08

**Authors:** Boshra Mozaffar, Arash Ardavani, Hisham Muzafar, Iskandar Idris

**Affiliations:** ^1^MRC-Versus Arthritis Centre for Musculoskeletal Ageing Research, National Institute for Health Research Nottingham Biomedical Research Centre, Clinical, Metabolic and Molecular Physiology, University of Nottingham, Royal Derby Hospital, Derby, UK; ^2^Clinical Nutrition Department, Applied Medical Sciences, Jazan University, Jazan, Saudi Arabia; ^3^Department of Pharmacology and Toxicology, College of Pharmacy, Jazan University, Jazan, Saudi Arabia

## Abstract

**Introduction:**

Food taste and flavour affect food choice and acceptance, which are essential to maintain good health and quality of life. Reduced circulating zinc levels have been shown to adversely affect the taste, but the efficacy of zinc supplementation to treat disorders of taste remains unclear. In this systematic review and meta-analysis, we aimed to examine the efficacy of zinc supplementation in the treatment of taste disorders.

**Methods:**

We searched four electronic bibliographical databases: Ovid MEDLINE, Ovid Embase, Ovid AMAD, and PubMed. Article bibliographies were also searched, which yielded additional relevant studies. There were no restrictions on the publication date to facilitate the collection and identification of all available and relevant articles published before 7 February 2021. We performed a systematic review and meta-analysis according to the PRISMA Statement. This review was registered at PROSPERO and given the identification number CRD42021228461.

**Results:**

In total, we included 12 randomized controlled trials with 938 subjects. The intervention includes zinc (sulfate, gluconate, picolinate, polaprezinc, and acetate), and the pooled results of the meta-analysis of subjects with idiopathic and zinc-deficient taste disorder indicate that improvements in taste disorder occurred more frequently in the experimental group compared to the control group (RR = 1.38; 95% CI: 1.16, 1.64, *p*=0.0002). Zinc supplementation appears to confer a greater improvement in taste perception amongst those with chronic renal disease using zinc acetate (overall RR = 26.69, 95% CI = 5.52–129.06, *p* < 0.0001). The doses are equivalent to 17 mg–86.7 mg of elemental zinc for three to six months.

**Conclusion:**

Zinc supplementation is an effective treatment for taste disorders in patients with zinc deficiency, idiopathic taste disorders, and in patients with taste disorders induced by chronic renal failure when given in high doses ranging from 68 to 86.7 mg/d for up to six months.

## 1. Introduction

Food taste and flavour are important elements that affect food choice and acceptance [1]. Disorders of taste can adversely affect patients' health and quality of life [2], resulting in loss of food enjoyment, poor appetite, unintended weight loss, malnutrition, and other psychological and physiological complications [3–5]. Taste disorder is characterised by unpleasant tastes, where patients can experience hypogeusia (a condition of reduced ability to taste sweet, sour, bitter, salty, and umami tastes) or ageusia (a total loss of the ability to detect tastes) or dysgeusia (persistent foul, salty, rancid, or metallic taste sensation in the mouth) [6]. Around 200,000 patients visit doctors each year in the US complaining of a change in either taste or smell [1]. In 2003, about 240,000 patients were diagnosed with taste disorders in Japan [2]. A recent US survey using the Chemical Senses Questionnaire (CSQ) reported that the prevalence of taste alteration was 19% in the adult population, with 5% reporting dysgeusia, reaching 27% in elderly populations [7]. More than half of patients (56.9%) in Italy with COVID-19 have reported a reduction of taste and/or smell; a severe reduction of taste was present in 39.7% of patients [8]. Taste alteration is also observed in 66% of chemotherapy patients [9]. The most common causes of taste disorder are medications (21.7%), followed by zinc deficiency (14.5%), oral and perioral infections, Bell's palsy, oral appliances and age while less common causes include nutritional factors, tumours or lesions associated with taste pathways, head trauma, exposure to toxic chemicals and radiation treatment of the head and neck [10].

Zinc is an important element that supports many functions in humans including the immune system, growth, and development [11]. In addition, zinc is important for the functioning of taste buds [12]. Disturbance of salivary zinc levels has been found to be associated with a decreased level of gustin [13]. Gustin is the major zinc-containing protein in the human parotid saliva [12]; decreases in the secretion of gustin have been linked with abnormalities of the growth and development of the taste buds and the resultant loss of taste [14]. This mechanism is supported by numerous studies finding that patients with hypogeusia had low levels of gustin and salivary zinc [14–16] as well as significant alterations in the shape of taste buds [15]. The association between zinc deficiency and taste disorders has been well known for years [17–19], but evidence for efficacious treatment for taste disorders in clinical practice remains lacking. Although taste disorder has not been given sufficient attention by the medical community and researchers, in recent years, increased interest has emerged in evaluating potential treatments for disorders of taste due to the increasingly recognised adverse effects affecting taste due to bariatric surgery [20] and most recently due to COVID-19 infections [21]. We, therefore, aim to perform a systematic literature review and meta-analysis for available randomized controlled trials to investigate the efficacy of zinc supplementation in the treatment of taste disorders in the adult population.

## 2. Methods

We performed our systematic review and meta-analysis according to the Preferred Reporting Items for Systematic Reviews and Meta-Analysis (PRISMA) statement [22] to identify the effectiveness of zinc supplementation to prevent and treat taste disorder in patients who had been diagnosed with zinc deficiency, idiopathic taste disorder, or taste disorder secondary to chronic renal failure. Included and excluded studies were assessed based on outcomes, participants, intervention types, and study types.

### 2.1. Inclusion and Exclusion Criteria

#### 2.1.1. Study Types

We only included randomized control trials; all other study designs were excluded.

#### 2.1.2. Participants

All included participants consisted of human populations, and animal studies were excluded. Participant groups consisting of adults ≥18 years were included. We excluded patients who received chemotherapy and radiation, children, and pregnant women. We also excluded patients with taste disorders induced by drug use or taste disorders induced by the common cold.

#### 2.1.3. Intervention

The participants received zinc-based therapy for the prevention and treatment of taste disorders compared to controls who received a placebo.

#### 2.1.4. Outcomes

Improvement of taste disorder in response to zinc treatment was observed in intervention groups compared to the control group at the baseline and during a follow-up period. Zinc levels were also compared before and after treatment. Papers that did not include zinc or taste change outcomes were excluded.

### 2.2. Search Strategy

A literature search was conducted to describe the effects of zinc supplementation to improve subjective and objective symptoms of taste disorder induced by zinc deficiency, idiopathic conditions, or chronic renal failure. Two authors conducted the systematic search in the following electronic bibliographical databases: Ovid MEDLINE, Ovid Embase, Ovid AMAD, and PubMed. Article bibliographies were also searched and yielded additional relevant studies. There were no restrictions on publication date, facilitating the collection and identification of all available and relevant articles published before 7 February 2021. The following keywords were used: “taste change,” “taste disorder,” “taste dysfunction,” “dysgeusia,” “zinc,” “zinc sulphates,” and “deficiency.” The systematic review was registered at PROSPERO (https://www.crd.york.ac.uk) and given the identification number CRD42021228461.

PubMed search strategies are as follows. (“taste disorders” (MeSH Terms) OR taste disorder (Text Word)) OR (“taste” (MeSH Terms) OR taste (Text Word)) AND change (All Fields)) OR (“taste” (MeSH Terms) OR taste (Text Word)) AND disfunction (All Fields)) OR (“dyspepsia” (MeSH Terms) OR dyspepsia (Text Word) AND (“zinc” (MeSH Terms) OR zinc (Text Word)) OR (“zinc” (MeSH Terms) OR zinc (Text Word)).

### 2.3. Data Extraction

We reviewed the articles according to the inclusion and exclusion criteria and summarised the main findings. Data regarding study duration, sample size, methods of detection of taste disorder, zinc dose, treatment period, and outcomes were extracted and are summarised in Tables 1 and 2. All the data those were utilised for the meta-analysis component were dichotomous data to find out the number of events in both the intervention and placebo groups. Additionally, all zinc supplement doses were considered for meta-analysis implementation.

### 2.4. Assessment of the Risk of Bias in Selected Studies

We used the Cochrane quality assessment tool to the assessed risk of bias for randomized controlled trials. The Cochrane tool, as described in the Handbook for Systematic Reviews of Interventions, evaluates the following attributes: random sequence generation (selection bias), allocation concealment (selection bias), blinding of participants and personnel (performance bias), blinding of outcome assessment (detection bias), incomplete outcome data (attrition bias), selective reporting (reporting bias), and other forms of bias. Rating criteria include low risk of bias, high risk of bias, or unclear risk of bias [35]. The Cochrane risk-of-bias tool for randomized trials (RoB) was independently performed by two investigators (BM and HM).

### 2.5. Statistical Procedures

The meta-analysis was conducted using Review Manager 5. The Mantel–Haenszel (M–H) statistical method was selected with the random effect method for dichotomous data and established the outcome measure as a total and event based on Cochrane recommendation. All pooled results were reported as relative risk (RR) and 95% confidence intervals (CI) for all individual studies, in addition to an effect size estimate (*Z*-statistic) and a measure of statistical significance (*p* < 0.05). To distinguish between the observed effects of zinc supplementation in iatrogenic or primary zinc deficiency versus chronic renal disease, two separate forest plots were generated for each. Further, data points from all studies at the synthesis stage were included, where data pertaining to event and total count, the equivalent quantity of elemental zinc, and the pharmaceutical name of the zinc supplement are stated. Finally, subanalysis was performed, based on the pharmaceutical name of the zinc supplement (s) included at the quantitative synthesis stage.

### 2.6. Assessment of Heterogeneity

We followed the Cochrane Handbook for Systematic Review of Interventions guidelines to assess the heterogeneity of the studies that were generated through the associated forest plots using Review Manager 5. Using the chi-squared test, we interpreted the heterogeneity according to *I*^2^ statistics: 75–100% indicates considerable heterogeneity, 50–90% represents substantial heterogeneity, 30–60% represents moderate heterogeneity, and 0–40% represents insignificant heterogeneity [35].

### 2.7. Summarizing and Interpreting Results

Review Manager 5 was used to conduct the meta-analysis, the risk-of-bias assessment, and the summary of the findings in Table 3 for each outcome included in this review. We imported the data to GRADEpro software to assess the evidence for each outcome. GRADE was also used to assess the quality of reported results in Table 4. We did not perform an analysis for publication bias via funnel plot as there were less than 10 studies included in the meta-analysis. This is because when there are fewer studies the power of the tests is too low to distinguish the chance from real asymmetry and in this study the largest forest plot only had seven data points across four studies.

## 3. Results

### 3.1. Study Selection

**Figure 1:**
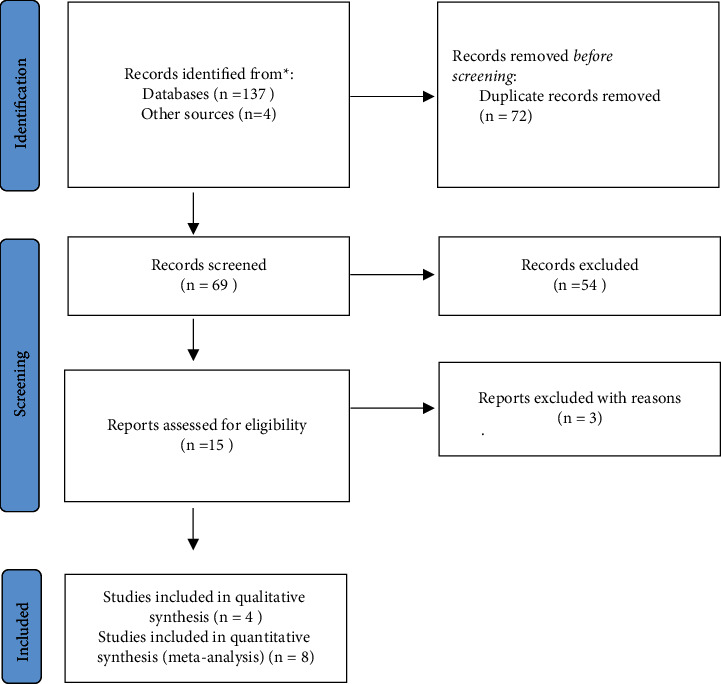
PRISMA flow diagram of the study selection and identification process. PRISMA, Preferred Reporting Items for Systematic Reviews and Meta-Analysis.

A flow diagram of our literature search is shown in Figure 1. Following exclusions and removals, complete data extraction was performed on a total of 12 articles that met the inclusion criteria. Of these studies, four were included in a qualitative synthesis, and eight were included in a quantitative synthesis (meta-analysis) [36]. The characteristics of these 12 articles are shown in Table 1.

### 3.2. Study Characteristics

#### 3.2.1. Trial Settings

Twelve randomized controlled trials (RCTs) are included in this review; all but one was written in English. One was in Japanese but was translated to English (Ikeda et al. [23]) The most common countries of origin of these studies were Japan and the US; one was from the UK, and one was from Germany. Out of 12 trials, 2 were crossover trials.

### 3.3. Study Populations

A total of 938 subjects were included in this study, all adults. The minimum age included in the trials was 18 years or older and the highest age observed was 84 years old; the lowest sample size was 22 and the highest sample size was 219. Eight studies included both genders in their trials; one study included only males and three trials did not report gender distribution.

Four studies were on idiopathic taste disorder, three concerned idiopathic and zinc-deficient taste disorder, and five were on renal failure-induced taste disorder.

### 3.4. Risk of Bias in Included Studies

Most studies were found to have an unclear risk of bias. However, four studies have a high risk of bias and three studies have a low risk of bias.

### 3.5. Intervention and Duration

#### 3.5.1. Idiopathic and Zinc-Deficient Taste Disorder

**Figure 2:**
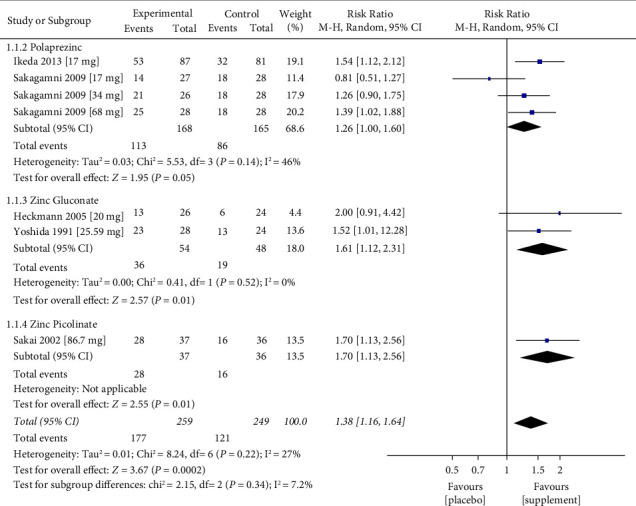
Meta-analysis of the effect of zinc replacement for the treatment of taste disorder. Forest plot including data analysis of five studies with a total of 508 cases of idiopathic and zinc-deficient taste disorder enrolled to experimental (*n* = 259) and control groups (*n* = 249). Data expressed as event “total number of cases that improved after receiving the treatment or placebo,” and total “total number of participants in either control or experimental group” *p* value for heterogeneity was 0.22. The pooled results of this meta-analysis indicated that taste disorder improvement occurred significantly more frequently in the supplemented group compared to the control group. Overall RR is positive (RR = 1.38, 95% CI = 1.16–1.64), and statistical significance was found to be *p*=0.0002.

*(1) Polaprezinc*. First, we evaluated the efficacy of polaprezinc supplementation in idiopathic and zinc-deficient taste disorders. The efficacy of polaprezinc was examined in two studies, using different dosages. Sakagami et al. [24] introduced three different dosages to the intervention group: 75 mg, 150 mg, and 300 mg, which are equivalent to 17 mg, 34 mg, and 68 mg of elemental zinc. Despite the utilisation of identical doses (17 mg), Ikeda et al. [23] and Sakagami et al. [24] presented with differing outcomes (RR = 1.54, 95% CI = 1.12–2.12, and RR = 0.81, 95% = 0.51–1.27, respectively) (Figure 2). Nonetheless, across the Polaprezinc subgroup data points from Sakagami et al. [24], an increase in effect size is observed (Figure 2). Although an overall supplement-specific RR is positive (RR = 1.26, 95% CI = 1.00–1.60), statistical significance was found to be borderline (*p*=0.05) (Figure 2).

*(2) Zinc Gluconate*. Three trials studied the efficacy of zinc gluconate supplementation in idiopathic and zinc-deficient taste disorders. Yoshida et al. [29] administered 158 mg of zinc gluconate (equivalent to 22.59 mg/d of elemental zinc) for four months at a high risk of bias. Heckmann et al. [26] administered 140 mg (equivalent to 20 mg of elemental zinc) for three months at low risk of bias. An improvement in taste disorder was observed for the zinc supplement groups (RR 1.61, 95% CI: 1.12–2.31, *p*=0.01) among 102 participants (Figure 2).

Stewart-Knox et al. [25] administered zinc gluconate equivalent to 15 or 30 mg of elemental zinc per day over six months and were at high risk of bias. The study showed that zinc level increased postintervention in both groups and were greater in the 30 mg supplemented group; acuity for salt taste was greater in the 30 mg supplemented group (*p*=0.031) while 15 and 30 mg Zn groups did not improve any tastes acuity. However, we could not conduct a meta-analysis of the results because the study did not report the number of events in the placebo group.

*(3) Zinc Picolinate*. Of the studies included, only one [28] was found to examine the efficacy of zinc picolinate on taste disorder patients at a high risk of bias. An improvement in taste disorder at a dosage of 28.9 mg three times/d for three months (RR 1.70, 95% CI: 1.13–2.56, *p*=0.01) (Figure 2), with 73 participants.

*(4) Zinc Sulphate*. In 1976, Henkin et al. [34] examined the effectiveness of four doses of 100 mg of zinc ion, with an unclear risk of bias. The results from this study indicated that both placebo and treatments groups with zinc sulfate showed equivalent improvements. We excluded this study from the meta-analysis because number of events in both the intervention and placebo groups was unclear.

#### 3.5.2. Zinc Disorder Secondary to Chronic Renal Failure

**Figure 3:**
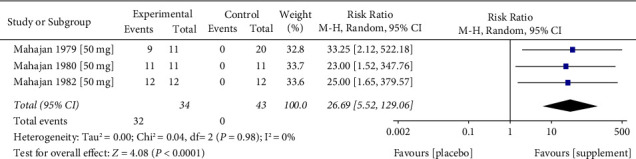
Meta-analysis of the effect of zinc replacement for taste disorder in patients with chronic renal failure. Forest plot including data analysis of three studies with a total of 77 cases of taste disorder induced by chronic renal failure, enrolled to experimental (*n* = 34) and control groups (*n* = 43). Data expressed as event “total number of cases that improved after receiving the treatment or placebo” and total “total number of participants in either control or experimental group” *p* value for heterogeneity was 0.98. The pooled results of this meta-analysis indicated that taste disorder improvement occurred significantly more frequently in the supplemented group compared to the control group. Overall RR is positive (RR = 26.69, 95% CI = 5.52–129.06), and statistical significance was found to be *p* < 0.0001.

*(1) Zinc Acetate*. Zinc acetate was used as a treatment for taste disorder induced by chronic renal failure in three studies [30–32]. Each study provided a single data point each, with the overall RR for zinc acetate found to be 26.69 (95% CI = 5.52–129.06, *p* < 0.0001) (Figure 3). The total number of participants in the three studies was 77 patients. A heterogeneity assessment was inconclusive (*I*^2^ = 0%, *p*=0.98) (Figure 3).

*(2) Zinc Sulphate*. Two studies, Atkin-Thor et al. [33] and Matson et al. [27], examined the efficacy of zinc sulfate in taste disorder induced by chronic renal failure for up to a six-week intervention period. In a double-blind crossover trial, Atkin-Thor et al. [33] introduced 440 mg of zinc sulfate three times per week at a high risk of bias, the results of this study showed a significant improvement in taste acuity in the supplemented group. Whereas Matson introduced 220 mg of zinc sulphate per day at an unclear risk of bias, the results from this study showed no improvements in both the intervention and placebo groups. These two trials did not provide sufficient details about the placebo groups. We have therefore excluded them from the meta-analysis.

## 4. Discussion

This systematic review assessed the efficacy of zinc supplementation to improve taste disorders. We focused on the outcomes of intervention groups compared to placebo among patients with zinc deficiency and idiopathic taste disorder or taste disorder induced by chronic renal failure. We included 12 randomized controlled trials: four were included in a qualitative synthesis and eight in a meta-analysis. We assessed five studies as having an unclear risk of bias [23, 24, 27, 32, 34], four studies at a high risk of bias [25, 28, 29, 33], and three studies at low risk of bias [26, 30, 31]. Seven included studies examined the effectiveness of different zinc supplementations (polapre zinc, picolinate, zinc gluconate, and zinc sulphate) among patients with zinc deficiency and idiopathic taste disorder. We did not include two studies such as the study by Henkin et al. [34] and Stewart-Knox et al. [25] in the meta-analysis because of their unclear methodologies and unreported data for the placebo groups. Out of seven studies that examined the efficacy of zinc supplementation in taste disorders induced by chronic renal failure, we did not include Atkin-Thor et al. [33] and Matson et al. [27] in the meta-analysis because they did not report data about the placebo groups.

### 4.1. Summary of Main Results

The pooled results of this meta-analysis indicated that improvement in taste disorder occurred significantly more frequently in the intervention group compared to the control group. There was a significant improvement in taste following zinc supplementation at the study level except in three studies [24, 26, 29]. The improvement in taste following zinc supplementation was observed at the meta-analysis level. We found that zinc supplements reduced the risk of taste disorder by 51%. Moreover, the pooled results of the largest studies [23, 24, 28] indicated that zinc supplementation is an effective treatment for taste disorders in patients with zinc deficiency or idiopathic taste disorders when given in high doses ranging from 68 to 86.7 mg/d for up to three months. This results in agreement with Yagi et al.'s [37] review which indicated that zinc supplementation contributes to the treatment of taste disorders caused by zinc deficiency. In contrast, Kumbargere Nagraj et al. [38] did not find sufficient trials to support the effectiveness of zinc in taste disorder improvement. The level of included studies ranged from moderate to high using The Grading of Recommendations Assessment, Development and Evaluation (GRADE). Heckmann et al. [26] and Yoshida et al. [29] introduced a low dose of elemental zinc, around 20–22.59 mg/d, for up to three to four months to patients with taste disorders induced by zinc deficiency or idiopathic disease and our meta-analysis showed insignificant improvement of taste disorders, however, the results for these two trials should be viewed with caution due the quality of evidence was rated as low, and high risk of bias for one study Yoshida et al. [29].

In the three studies concerning taste disorder induced by chronic renal failure, we found the level of evidence and its quality to be low. This was driven by the fact that the studies mainly had small sample size and the absence of event numbers in the placebo group, which resulted in a high upper limit of the CI [30–32] in the meta-analysis. Overall, per the available data, zinc supplementation appears to confer a greater improvement in taste perception amongst those with chronic renal disease using zinc acetate (overall RR = 26.69, 95% CI = 5.52–129.06, *p* < 0.0001) (Figure 3) in comparison to the extent of improvement using alternative supplements in the iatrogenic or zinc deficiency disease groups (Figure 2). Unfortunately, a direct comparison in the response to zinc acetate between the chronic renal disease and iatrogenic or zinc deficiency cohorts was not possible due to missing data. Furthermore, zinc picolinate was represented by a single data point [28]. In all studies included in this meta-analysis, we did not find considerable statistical heterogeneity. Nevertheless, there is substantial heterogeneity based on elemental zinc-equivalent dose, supplement chemical structure, follow-up time, and disease state exists, as inferred based on the study characteristics as we aimed to collect all available RCTs to examine the effectiveness of zinc supplementation in taste disorder treatment. We suggest that zinc supplementation may improve specific tastes more than others depending on the case or the disease-induced taste disorder. We suggest a high dose of elemental zinc 68–86.7 mg/d for up to six months to improve taste disorders. However, the results of this meta-analysis should be interpreted with caution as excessive zinc supplementation might have serious health outcomes and toxicity when taken at a significantly higher than the Recommended Dietary Allowance (RDA) (100–300 mg/day vs. 15 mg daily). It has been proposed that even smaller doses of zinc supplementation, closer to the RDA, interfere with the utilisation of copper and iron and negatively impact HDL cholesterol levels. Zinc supplement users should be informed of any potential risks associated with its usage [39].

### 4.2. Strengths and Limitations of This Study

Unlike other reviews in this area, our systematic review provided additional evidence and clarification of zinc supplementation's efficacy in improving taste disorder in adult populations by stratifying according to zinc dose, formulation type, and treatment duration. However, one aspect that can limit the analysis and discussion of the results is the heterogeneity of the methods used. The studies assessed combined objective outcomes (e.g., filter paper disk; detection and recognition thresholds for sweet, sour, salty, bitter, and umami tastes) and subjective outcomes (e.g., questionnaires results). However, whether the difference between subjective and objective methods could significantly affect the results of improvement is unclear. In another review, the author examined the overall improvement in taste acuity using both subjective and objective methods; however, the author could not conclude the overall effect because of the very low level of evidence. High-quality research is required to compare different objective and subjective methods [38]. We observed that some studies detected taste improvement in only one type of taste, so a further limitation of our meta-analysis is that we defined “improvement” as an improvement of any of the five basic tastes: sweet, sour, bitter, salty, and umami tastes.

## 5. Conclusion

High-dose zinc supplementation is an effective treatment for taste disorders in patients with zinc deficiency or idiopathic taste disorder and in patients with taste disorders induced by chronic renal failure.

## Figures and Tables

**Table 1:** The main patient characteristics of the included studies.

Author (s), year (study duration)	Country	Study type	Total no. of patients	No. of patients receiving zinc	No. of patients receiving placebo	Gender	Age (year)	Zinc supplement	Disease or case-induced taste disorder
Ikeda et al., 2013 [23] (12 weeks)	Japan	Double-blind RCT w/placebo	219	108	111	87 M and 132 F	Average for intervention: 43.3 Control: 47.1	Polaprezinc, 34 mg/d	Zinc deficiency and idiopathic taste disorder
Sakagami et al., 2009 [24] (12 weeks)	Japan	Double-blind RCT w/placebo. Multicentre	109	81	28	M and 56 F 51	20–80	Polaprezinc, (group 1) 17 mg (*n* = 27), (group 2) 34 mg (*n* = 26) or (group 3) 68 mg (*n* = 28) daily	Idiopathic taste disorder
Stewart-Knox et al., 2007 [25] (6 months)	Japan	Double-blind RCT w/placebo	199	NR	NR	103 M and 96 F	70–78	Zinc gluconate, elemental zinc gluconate (group 1) 15 mg/d and (group 2) 30 mg/d	Idiopathic taste disorder in elderly people
Heckmann et al., 2005 [26] (3 months)	Germany	RCT w/placebo	50	24	26	7 M and 43 F	41–82	Zinc gluconate (140 mg/d, equivalent to 20 mg/d of elemental zinc)	Idiopathic taste disorder
Matson et al., 2003 [27] (6 weeks)	UK	Double-blind RCT w/placebo	24	12	12	M and F	30 to 72	Zinc sulphate	Chronic renal failure
Sakai et al., 2002 [28] (3 months)	Japan	Double-blind RCT w/placebo	73	37	36	M/NR and 47 F	23–79	Picolinate, 28.9 mg of elemental zinc three times/d	Zinc deficiency and idiopathic taste disorder
Yoshida et al., 1991 [29] (4 months)	Japan	Double-blind RCT w/placebo	52	28	24	M to F ratio was 1 : 1.8	Mean age for group 55.1 & 59.2 for placebo	Zinc gluconate, 158 mg zinc gluconate; zinc content: 22.59 mg	Zinc deficiency and idiopathic taste disorder
Mahajan et al., 1982 [30] (6 months)	USA	Double-blind RCT w/placebo	24	12	12	Only males	Treatment 46 ± 8 Control 49 ± 12	Zinc acetate, zinc acetate (50 mg of elemental zinc/d)	Chronic renal failure
Mahajan et al., 1980 [31] (6 months)	USA	Double-blind RCT w/placebo	22	11	11	NR	Treatment: 51.3 ± 3.2 Control: 55.1 ± 2.8	Zinc acetate, zinc acetate (50 mg of elemental zinc/d)	Chronic renal failure
Mahajan et al., 1979 [32] (6 months)	USA	Double-blind RCT w/placebo	31	11	20	NR	NR	Zinc acetate, zinc acetate (50 mg of elemental zinc/d)	Chronic renal failure
Atkin-Thor et al., 1978 [33] (6 weeks)	USA	Double-blind crossover RCT w/placebo	29	20	9	NR	21–70	Zinc sulphate, 440 mg ZnSO_4_ postdialysis, 3 times per week	Chronic renal failure
Henkin et al., 1976 [34] (6 months)	USA	Double-blind crossover RCT w/placebo	106	Not clear	Not clear	53 M and 53 F	19–84	Zinc sulphate, 100 mg of zinc ion in four oral doses	Idiopathic taste disorder

NR: not reported, M: male, F: female, RCT: randomized controlled trial, w: with.

**Table 2:** Summary of the findings of the included studies.

Author (s), year	Test method	Statistical analysis	Zinc level at baseline	Zinc status after treatment/intervention group	Zinc status after treatment/placebo group	Significant improvement against placebo	Taste status/intervention group	Taste status/placebo group	Significant improvement against placebo
Ikeda et al., 2013 [23]	Filter paper disc method	Wilcoxon rank-sum test and Fisher's exact test	71.1 *µ*g/dl zinc group, 73.5 *µ*g/dl placebo group	NR	NR	NR	NR	NR	NR
Sakagami et al., 2009 [24]	Filter paper disc method and serum zinc level	Shirley-Williams test and unpaired student's *t*-test, Dunnett's test, Fisher's exact test	↓ 69 *µ*g/dl	Serum zinc level ↑ 5.7 ± 13.5 (17 mg), 11.4 ± 16.6 (34 mg), and 20.6 ± 21.3 (68 mg), respectively, (*p* < 0.001), statistically significant increase in group receiving 68 mg of zinc (*p* < 0.001)	1.8 ± 12.7	(*p* < 0.001) sig	The efficient rate of gustatory sensitivity was 51.9%, 80.0% and 89.3% in the 17 mg, 34 mg and 68 mg zinc-treated groups, respectively	The efficient rate (63.0%)	*p*=0.018 sig
Stewart-Knox et al., 2008 [25]	Detection thresholds for sweet, sour, salty, bitter and umami	Factorial ANOVA	Within normal range for placebo and zinc groups (11–18 *µ*·mol/l)	Zinc level increased postintervention in both groups and greater in the 30 mg group	13.05 ± 1.66 *µ*·mol/l	*p*=0.000 sig	Acuity for salt taste was greater in the 30 mg supplemented group (0·84 409 (SD 0·13 349) 15 and 30 mg Zn did not improve any tastes acuity	0·75 045 ± 0·210	*p*=0.031 sig
Heckmann et al., 2005 [26]	Filter paper strips and serum zinc level	*T*-test for independent samples and correlation (Pearson's) test	72.78 ± 18.38 mg/dl	No significant change in serum zinc level before and after treatments. 81.53 ± 19.61	72.01 ± 10.22	*p*=0.65 not sig	There were significant improvements in the zinc group compared to the placebo group 25.7 ± 6.5	21.2 ± 5.7	*p* < 0.001 sig
Matson et al., 2003 [27]	Filter paper disc method, questionnaire and serum zinc level	Unpaired *t*-test	Placebo 10.9 ± 1.1_ *µ*·mol/l Intervention 9.9 ± 1.6 *µ*·mol/l	Intervention 10.4 ± 1.4 *µ*·mol/l	Placebo 10.5 ± 1.6 *µ*·mol/l	NS	Not improved	Not improved	NS
Sakai et al., 2002 [28]	A questionnaire, filter paper disc method and serum zinc level	Student's *t*-test and Wilcoxon's test	For zinc-deficient group ≤69 *µ*g/dl, idiopathic (70 *µ*g/dl ≥	Significantly greater increase in serum zinc level after treatment 81.6 *µ*g/dl	Serum zinc level after treatment 72.3 *µ*g/dl	*p* < 0.01 sig	There was a significant difference between groups in objective improvements in taste 28 patients improved and 9 not improved	16 improved and 20 not improved	*p* < 0.01 sig
Yoshida et al., 1991 [29]	Filter paper disc method and serum zinc level	Chi-square test	For zinc-deficient group, 60–69 *µ*g/dl; for idiopathic group, 70 *µ*g/dl or higher	Serum zinc concentration was significantly higher during the intervention period, 94.0 ± 24.6 (*n* = 20)	71.9 ± 10.9 (*n* = 23)	*p* < 0.01 sig	A significant difference was detected between the two groups in therapeutic efficiency (23 patients improved and 5 unchanged)	13 patients improved and 11 unchanged	*p* < 0.05 sig
Mahajan et al., 1982 [30]	The threshold of taste detection and recognition for salty, sweet and bitter, and serum zinc level	Paired student's *t*-test	Intervention 81 ± 8 *µ*g/dl Placebo 82 ± 6 *µ*g/dl	110 ± 14 *µ*g/dl	84 ± 9 *µ*g/dl	*p* < 0.005 sig	Improved	Not improved	*p* < 0.05 sig
Mahajan et al., 1980 [31]	The threshold of taste detection and recognition for salty, sweet and bitter, and serum zinc level	Independent student's *t*-test for unpaired data	Serum zinc level in treatment group 70 *µ*g/dl, lower than the control group	The mean plasma zinc level increased significantly from 75 ± 8 to 97 ± 10 *µ*g/dl	No change (75 ± 15 to 80 ± 15)	*p* < 0.001 sig	Significant improvement in sweet, salty, and bitter taste	Not improved	*p* < 0.05 sig
Mahajan et al., 1979 [32]	The threshold of taste detection and recognition for salty, sweet and bitter, and serum zinc level	Independent student's *t*-test for unpaired data	75.0 ± 2.0 *µ*g/dl zinc group	97.2 ± 3.2 *µ*g/dl zinc group	NR	Sig	Improved	Not improved	*p* < 0.005 sig
Atkin-Thor et al., 1978 [33]	The threshold of taste detection and recognition for salty, sweet and bitter, and zinc concentration in hair	NR	Zinc concentration in hair before treatment is 2 (10%) where the normal range 180 ± 4 ppm (100%)	The serum zinc level was not published in the study; however, zinc concentration in hair increased in 85% of patients	NR	*p* < 0.01 sig	Significant improvement in taste acuity in the supplemented group by 95% of patients 6 weeks after treatment	NR	*p* < 0.01 sig
Henkin et al., 1976 [34]	The threshold of taste detection and recognition for salty, sweet and bitter measured with the total concentration of zinc and copper and a questionnaire of taste acuity	Student's *t*-test	NR	NR	NR	NR	Significant improvement in taste disorder at 3 months	NR	NR

**Table 3:** Summary of findings.

Zinc supplement compared to placebo for improvement of taste disorder						
Patient or population: improvement of taste disorder						
Intervention: zinc supplement						
Comparison: placebo						
Outcomes	Anticipated absolute effects^*∗*^ (95% CI)		Relative effect (95% CI)	No. of participants (studies)	Certainty of the evidence (GRADE)	Comments
	Risk with placebo	The risk with zinc supplement				
Polaprezinc supplementation in idiopathic and zinc-deficient taste disorder patients, equivalent to (17 mg, 68 mg) of elemental zinc for 12 weeks	454 per 1,000	671 per 1,000 (535 to 839)	RR 1.48 (1.18 to 1.85)	223 (2 RCTs)	⊕⊕⊕⊕ HIGH	Polarprezinc supplementation improved taste disorder in idiopathic and zinc-deficient patients compared with the placebo by 48%, with a CI of 18% to 85% increase in taste acuity
Zinc gluconate supplementation in idiopathic and zinc-deficient taste disorder patients, equivalent to (20 mg, 22.59 mg) per day of elemental zinc for 3-4 months	396 per 1,000	637 per 1,000 (443 to 914)	RR 1.61 (1.12 to 2.31)	102 (2 RCTs)	⊕⊕○○ LOW^a,b^	Zinc gluconate supplementation improved taste disorder in idiopathic and zinc-deficient patients compared with the placebo by 61% with a CI of 12% to 131% increase in taste acuity
Zinc picolinate supplementation in idiopathic and zinc-deficient taste disorder patients, equivalent to (28.9 mg) of elemental zinc three times per day for 3 months	444 per 1,000	756 per 1,000 (502 to 1,000)	RR 1.70 (1.13 to 2.56)	73 (1 RCT)	⊕⊕⊕○ MODERATE^c^	Zinc picolinate supplementation improves taste disorder in idiopathic and zinc-deficient patients compared with the placebo by 70% with a CI of 13% to 156% increase in taste acuity
Zinc acetate supplementation in patients with taste disorder induced by chronic renal failure, equivalent to (50 mg) per day of elemental zinc for 6 months	0 per 1,000	0 per 1,000 (0 to 0)	RR 26.69 (5.52 to 129.06)	77 (3 RCTs)	⊕⊕○○ LOW^d^	Zinc acetate supplementation improved taste disorder in patients with taste disorder induced by chronic renal failure compared with the placebo by 25.69% with a CI of 452% to 128.06% increase in taste acuity

^*∗*^The risk in the intervention group (and its 95% confidence interval) is based on the assumed risk in the comparison group and the relative effect of the intervention (and its 95% CI). CI: confidence interval; RR: risk ratio. GRADE working group grades of evidence. High certainty: We are very confident that the true effect lies close to that of the estimate of the effect. Moderate certainty: We are moderately confident in the effect estimate. The true effect is likely to be close to the estimate of the effect, but there is a possibility that it is substantially different. Low certainty: Our confidence in the effect estimate is limited. The true effect may be substantially different from the estimate of the effect. Very low certainty: We have very little confidence in the effect estimate. The true effect is likely to be substantially different from the estimate of effect. a: some concern with random sequence generation and lack of follow-up; b: wide confidence intervals in Heckmann et al. [26]; c: some concern of lack of follow-up; d: very wide confidence intervals in all three included trials.

**Table 4:** A systematic review meta-analysis—GRADE score results for all.

Lead author	Publication date	Risk of bias	Imprecision	Inconsistency	Indirectness	Publication bias
Ikeda et al. [23]	2013	⊕⊕⊕	No CI reported	⊕⊕⊕⊕	⊕⊕⊕⊕	N/A^*∗*^
Sakai et al. [28]	2002	⊕⊕	No CI reported	N/A	⊕⊕⊕	N/A^*∗*^
Yoshida et al. [29]	1990	⊕⊕	No CI reported	⊕⊕⊕⊕	⊕⊕⊕	N/A^*∗*^
Henkin et al. [34]	1976	⊕⊕⊕	No CI reported	N/A	N/A	N/A^*∗*^
Sakagami et al. [24]	2009	⊕⊕⊕	No CI reported	⊕⊕⊕⊕	⊕⊕⊕⊕	N/A^*∗*^
Heckmann et al. [26]	2005	⊕⊕⊕⊕	No CI reported	⊕⊕⊕⊕	⊕⊕⊕	N/A^*∗*^
Stewart-Knox et al. [25]	2008	⊕⊕	No CI reported	N/A	N/A	N/A^*∗*^
Mahajan et al. [32]	1979	⊕	No CI reported	⊕⊕⊕⊕	⊕⊕⊕⊕	N/A^*∗*^
Mahajan et al. [30]	1982	⊕⊕⊕⊕	No CI reported	⊕⊕⊕⊕	⊕⊕⊕⊕	N/A^*∗*^
Mahajan et al. [31]	1980	⊕⊕⊕⊕	No CI reported	⊕⊕⊕⊕	⊕⊕⊕	N/A^*∗*^
Atkin-Thor et al. [33]	1978	⊕⊕	No CI reported	⊕⊕	⊕⊕	N/A^*∗*^
Matson et al. [27]	2003	⊕⊕⊕	No CI reported	⊕⊕	⊕⊕⊕⊕	N/A^*∗*^

*Notes*. N/A-not a systematic review. N/A-not applicable, CI‐confidence interval. High: ⊕⊕⊕⊕, Moderate: ⊕⊕⊕, Low: ⊕⊕, Very Low: ⊕.

## Data Availability

The data used to support the findings of this study are available from the corresponding author upon reasonable request.

## References

[1] Clark J. E. (1998). Taste and flavour: their importance in food choice and acceptance. *Proceedings of the Nutrition Society*.

[2] Alvarez-Camacho M., Gonella S., Ghosh S. (2016). The impact of taste and smell alterations on quality of life in head and neck cancer patients. *Quality of Life Research*.

[3] Malaty J., Malaty I. A. C. (2013). Smell and taste disorders in primary care. *American Family Physician*.

[4] Hummel T., Basile N. L., Karl-Bernd H. (2018). The impact of smell and taste disorders. *GMS Current Topics in Otorhinolaryngology, Head and Neck Surgery*.

[5] Hur K., Choi J. S., Zheng M., Shen J., Wrobel B. (2018). Association of alterations in smell and taste with depression in older adults. *Laryngoscope Investigative Otolaryngology*.

[6] Nidcd (2021). Taste disorders. https://www.nidcd.nih.gov/health/taste-disorders.

[7] Rawal S., Hoffman H. J., Bainbridge K. E., Huedo-Medina T. B., Duffy V. B. (2016). Prevalence and risk factors of self-reported smell and taste alterations: results from the 2011–2012 US national health and nutrition examination survey (NHANES). *Chemical Senses*.

[8] Mercante G., Ferreli F., De Virgilio A. (2020). Prevalence of taste and smell dysfunction in coronavirus disease 2019. *JAMA Otolaryngol Head Neck Surg*.

[9] Campagna S., Gonella S., Sperlinga R. (2018). Prevalence, severity, and self-reported characteristics of taste alterations in patients receiving chemotherapy. *Oncology Nursing Forum*.

[10] Imoscopi A., Inelmen E. M., Sergi G., Miotto F., Manzato E. (2012). Taste loss in the elderly: epidemiology, causes and consequences. *Aging Clinical and Experimental Research*.

[11] Roohani N., Hurrell R., Kelishadi R., Schulin R. (2013). Zinc and its importance for human health: an integrative review. *Journal of Research in Medical Sciences*.

[12] Henkin R. I. (1984). Zinc in taste function: a critical review. *Biological Trace Element Research*.

[13] Brennan F., Stevenson J., Brown M. (2020). The pathophysiology and management of taste changes in chronic kidney disease: a review. *Journal of Renal Nutrition*.

[14] Henkin R. I., Martin B. M., Agarwal R. P. (1999). Decreased parotid saliva gustin/carbonic anhydrase VI secretion: an enzyme disorder manifested by gustatory and olfactory dysfunction. *The American Journal of the Medical Sciences*.

[15] Shatzman A. R., Henkin R. I. (1981). Gustin concentration changes relative to salivary zinc and taste in humans. *Proceedings of the National Academy of Sciences*.

[16] Zhu Y., Feron G., Von Koskull D., Neiers F., Brignot H., Hummel T. (2021). The association between changes of gustatory function and changes of salivary parameters: a pilot study. *Clinical Otolaryngology*.

[17] Ikeda M. I., Ikui A., Komiyama A., Kobayashi D., Tanaka M. (2008). Causative factors of taste disorders in the elderly, and therapeutic effects of zinc. *Journal of Laryngology and Otology*.

[18] Matsugasumi M., Hashimoto Y., Okada H. (2018). The association between taste impairment and serum zinc concentration in adult patients with type 2 diabetes. *Canadian Journal of Diabetes*.

[19] Yagi T. (2013). The role of zinc in the treatment of taste disorders. *Recent Patents on Food, Nutrition and Agriculture*.

[20] Ahmed K., Penney N., Darzi A., Purkayastha S. (2018). Taste changes after bariatric surgery: a systematic review. *Obesity Surgery*.

[21] Cazzolla A. P., Lovero R., Lo Muzio L. (2020). Taste and smell disorders in COVID-19 patients: role of interleukin-6. *ACS Chemical Neuroscience*.

[22] Page Mj M. J., Bossuyt P. M., Boutron I., Hoffmann T. C., Mulrow C. D. (2021). The PRISMA 2020 statement: an updated guideline for reporting systematic reviews. *International Journal of Surgery*.

[23] Ikeda M., Kurono Y., Inokuchi A. (2013). The effect of zinc agent in 219 patients with zinc deficiency-inductive/idiopathic taste disorder: a placebo controlled randomized study. *Nippon Jibiinkoka Gakkai Kaiho*.

[24] Sakagami M. I., Ikeda M., Tomita H. (2009). A zinc-containing compound, Polaprezinc, is effective for patients with taste disorders: randomized, double-blind, placebo-controlled, multi-center study. *Acta Oto-Laryngologica*.

[25] Stewart-Knox B. J. S., Simpson E. E., Parr H. (2008). Taste acuity in response to zinc supplementation in older Europeans. *British Journal of Nutrition*.

[26] Heckmann S. M., Hujoel P., Habiger S. (2005). Zinc gluconate in the treatment of dysgeusia--a randomized clinical trial. *Journal of Dental Research*.

[27] Matson A. W., Wright M., Oliver A. (2003). Zinc supplementation at conventional doses does not improve the disturbance of taste perception in hemodialysis patients. *Journal of Renal Nutrition*.

[28] Sakai F. Y., Yoshida S., Endo S., Tomita H. (2002). Double-blind, placebo-controlled trial of zinc picolinate for taste disorders. *Acta Oto-Laryngologica*.

[29] Yoshida S. E., Endo S., Tomita H. (1991). A double-blind study of the therapeutic efficacy of zinc gluconate on taste disorder. *Auris Nasus Larynx*.

[30] Mahajan S. K., Prasad A. S., Rabbani P., Briggs W. A., McDonald F. D. (1982). Zinc deficiency: a reversible complication of uremia. *The American Journal of Clinical Nutrition*.

[31] Mahajan S. K., Prasad A. S., Lambujon J., Abbasi A. A., Briggs W. A., McDonald F. D. (1980). Improvement of uremic hypogeusia by zinc: a double-blind study. *The American Journal of Clinical Nutrition*.

[32] Mahajan S. K., Prasad A. S., Lambujon J., Abbasi A. A., Briggs W. A., McDonald F. D. (1979). Improvement of uremic hypogeusia by zinc. *ASAIO Journal*.

[33] Atkin-Thor E., Goddard B. W., O’Nion J., Stephen R. L., Kolff W. J. (1978). Hypogeusia and zinc depletion in chronic dialysis patients. *The American Journal of Clinical Nutrition*.

[34] Henkin R. I., Schecter P. J., Friedewald W. T., Demets D. L., Raff M. (1976). A double blind study of the effects of zinc sulfate on taste and smell dysfunction. *The American Journal of the Medical Sciences*.

[35] Higgins T. J., Chandler J., Cumpston M., Li T., Page M. J., Welch V. A. (2019). *Cochrane Handbook for Systematic Reviews of Interventions*.

[36] Page M. J., McKenzie J. E., Bossuyt P. M. (2021). The PRISMA 2020 statement: an updated guideline for reporting systematic reviews. *International Journal of Surgery*.

[37] Yagi T., Asakawa A., Ueda H., Ikeda S., Miyawaki S., Inui A. (2013). The role of zinc in the treatment of taste disorders. *Recent Patents on Food, Nutrition and Agriculture*.

[38] Kumbargere Nagraj S., George R. P., Shetty N., Levenson D., Ferraiolo D. M., Shrestha A. (2017). Interventions for managing taste disturbances. *Cochrane Database of Systematic Reviews*.

[39] Fosmire G. J. (1990). Zinc toxicity. *The American Journal of Clinical Nutrition*.

